# Visual sensory stimulation interferes with people’s ability to echolocate object size

**DOI:** 10.1038/s41598-017-12967-3

**Published:** 2017-10-12

**Authors:** L. Thaler, D. Foresteire

**Affiliations:** Department of Psychology, Durham University, Durham, United Kingdom

## Abstract

Echolocation is the ability to use sound-echoes to infer spatial information about the environment. People can echolocate for example by making mouth clicks. Previous research suggests that echolocation in blind people activates brain areas that process light in sighted people. Research has also shown that echolocation in blind people may replace vision for calibration of external space. In the current study we investigated if echolocation may also draw on ‘visual’ resources in the sighted brain. To this end, we paired a sensory interference paradigm with an echolocation task. We found that exposure to an uninformative visual stimulus (i.e. white light) while simultaneously echolocating significantly reduced participants’ ability to accurately judge object size. In contrast, a tactile stimulus (i.e. vibration on the skin) did not lead to a significant change in performance (neither in sighted, nor blind echo expert participants). Furthermore, we found that the same visual stimulus did not affect performance in auditory control tasks that required detection of changes in sound intensity, sound frequency or sound location. The results suggest that processing of visual and echo-acoustic information draw on common neural resources.

## Introduction

Echolocation is the ability to use sound reverberation to obtain information about the distal spatial environment. It has long been established that certain species of bats and marine mammals use echolocation to navigate and locate prey^1^. Several studies have also demonstrated that humans are capable of using echolocation^2–4^. One of the most useful aspects of human echolocation is that it provides people a supplementary way of sensing their environment in low-vision conditions (e.g. permanent or temporary blindness). For example, echolocation can be used to distinguish various properties of silent distal objects, such as shape, size, distance, location or movement^2–4^.

Both blind and sighted people can learn to echolocate provided they have normal hearing^5,6^. Although individual performance varies considerably amongst sighted people^7,8^, on average, blind people outperform sighted people^2^. Blind people are also more sensitive to acoustic reverberations, even when they do not echolocate^9,10^.

Neuroimaging research suggests that in blind echolocators it is not only auditory, but also visual areas, which are involved in the processing of echoes^4^. Comparison of brain activity, between echo-acoustic and control sounds, and between different types of echo-acoustic sounds, highlight the involvement of calcarine cortex when blind echolocators processed echo-acoustic information^11–15^. This same area of the brain is known to be involved in visual processing in sighted people, suggesting that echolocation in blind people could be thought of as ‘seeing’ with sound. It is well known that blindness is associated with numerous changes on the behavioural and neural level^16–23^. Thus, the question arises if calcarine cortex activity associated with echolocation is limited to blind echolocators, or if involvement of calcarine cortex could also be relevant for echolocation in sighted people. To date, no study has found echolocation in sighted people to be related to activity in calcarine cortex. Critically, however, differences in brain activation were always coupled with performance differences in that participants with lower performance also showed less, or in some cases no, echo-related activity in calcarine cortex. Thus, there is the possibility that calcarine cortex activation in sighted people went undetected with fMRI due to a lack of performance and/or statistical power.

Drawing from behavioural findings in blind people, we propose that a functional link between echolocation and vision may indeed exist. People who are blind from birth show a deficit in the spatial calibration of external ‘allocentric’ space as compared to sighted people^24,25^. Yet, people who are blind from birth, but who also echolocate, perform just as well as sighted people and better than blind people who do not echolocate^26^. This suggests that echolocation may substitute vision for the calibration of external space. Further, blind people with expertise in echolocation are susceptible to an echo-acoustic illusion of size and weight which has previously only been reported in sighted people relying on visual cues to size^27^. Blind people who do not use echolocation do not show this illusory effect.

The current research tests the idea of a functional link between vision and echolocation in sighted people. To this end, we investigated whether visual stimulation would interfere with echolocation and reduce sighted, echolocation-naïve participants’ ability to echolocate object size. We measured participants’ performance when they echolocated in the absence of visual input and compared this to their performance when they echolocated under exposure to an uninformative visual stimulus (i.e. white light). If echolocation relied on the same neural networks necessary for visual processing, then ‘loading’ these areas with visual input should decrease participants’ performance^28^.

To distinguish task-specific sensory effects from attentional effects, we replicated our experiment and replaced the visual stimulus with a tactile stimulus (i.e. transcutaneous nerve stimulation). Presuming that there is no functional link between this form of tactile perception and echolocation, we predicted that the tactile stimulus would not interfere with echolocation. In addition to sighted participants, three blind expert echolocators participated in this tactile-echolocation condition to investigate whether effects in sighted people generalize to this special population. A pre-cursor visual-tactile matching experiment allowed us to establish the appropriate intensity level(s) for the tactile stimulus. To further address the issue of attentional effects, and to distinguish them from effects specific to the relationship between visual stimulation and echolocation, we conducted non-echolocation auditory control experiments. Using the same visual stimulus as in the main experiment, we combined it with auditory perception tasks in which participants were asked to detect changes in sound intensity or frequency, or in sound location. Presuming that there should be no functional relationship between visual stimulation and these auditory tasks, we predicted no significant change in participants’ performance.

We found that our results were in agreement with our hypothesis, suggesting that processing of visual and echo-acoustic information draw on common neural resources.

In subsequent sections we first present the methods for all experiments, followed by the results and discussion.

## Methods

All procedures were approved by the ethics board in the Department of Psychology at Durham University, and all methods were performed in accordance with the relevant guidelines and regulations laid out by the WMA in the declaration of Helsinki and the BPS code of practice. Blind participants were given accessible versions of all documents. We obtained written informed consent from all participants.

### Main Experiment – Effects of Visual and Tactile Sensory Stimulation on Echolocation of Size

To measure participants’ echolocation ability we used a paradigm introduced by Teng and colleagues^7^. This paradigm requires participants to use mouth-click based echolocation to detect the larger of two disks presented simultaneously. Visual or tactile sensory stimulation could be either ‘on’ or ‘off’ during the task.

### Participants

A total of 44 sighted, echo-naive people as well as 3 blind people with expertise in echolocation participated. This sample of sighted (and blind echo expert) participants was larger compared to previous research using this echolocation task^7,8^. All participants reported to have normal hearing and no history of any hearing difficulties. With respect to sighted, echo-naive participants (n = 44), 22 took part in the visual condition (5 male; mean age: 21.2; min: 18; max: 55; SD: 7.6), and 22 in the tactile condition (6 male; mean age: 22.2; min:18; max: 56; SD: 7.9). All sighted participants had normal or corrected to normal vision, and reported to not have prior experience with echolocation. Blind participants were totally blind at time of testing and reported using mouth-click based echolocation on a daily basis. (B1: male, 49 years at time of testing; enucleated in infancy because of retinoblastoma; reported to have used echolocation as long as he can remember. B2: male, 31 years at time of testing; lost sight gradually from birth due to Glaucoma. Since early childhood (approx 3 yrs) only bright light detection; reported to have used echolocation on a daily basis since he was 12 years old. B3: male, 33 years at time of testing; lost sight aged 14 years due to optic nerve atrophy; reported to have used echolocation on a daily basis since he was 15 years old). Blind participants only took part in the tactile condition. Participants volunteered to take part in the study and were compensated £7.50/hour or with participant pool credit.

### Set-Up and Apparatus

The experiment was conducted in a sound-insulated and echo-acoustic dampened room (approx. 2.9 m × 4.2 m × 4.9 m, noise-insulated room-inside-a-room construction, lined with acoustic foam wedges that effectively absorb frequencies above 315 Hz; noise floor 24 dBA). Participants were seated in the centre of the room on a height-adjustable chair.

#### Echolocation

**Figure 1:**
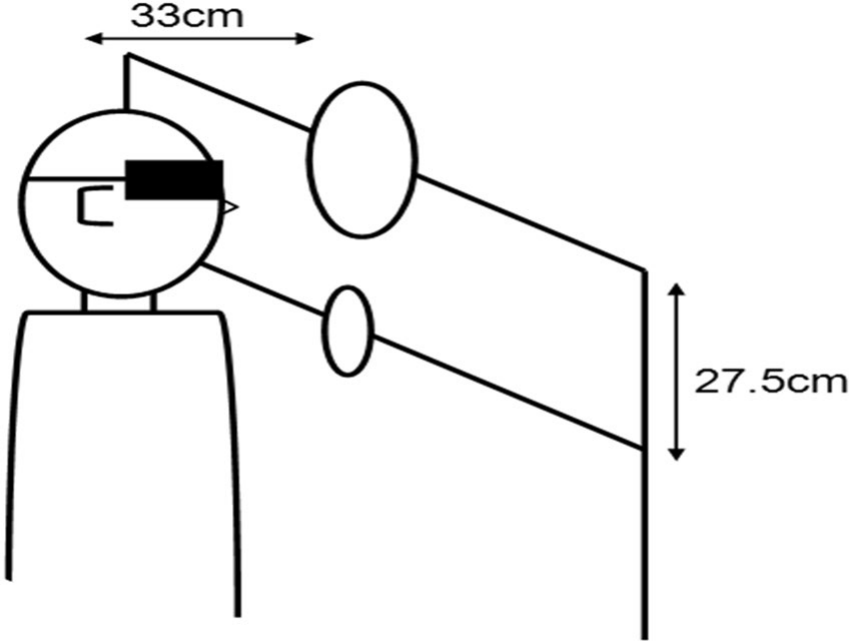
Apparatus used in the echolocation task. The participant’s task was to determine if the larger disk (reference disk) was on the bottom or top bar.

To measure participants’ echolocation ability we used an apparatus illustrated in Fig. 1, which was placed 33 cm in front of the participant. This apparatus is a replication of the apparatus used by^7^. The apparatus consisted of a frame made of metal rods (0.5 cm circular diameter). The frame stood up vertically and had two horizontal crossbars which were spaced 27.5 cm apart. The crossbars were used to mount flat, circular disks made from 0.5 cm thick acrylic. The disks were mounted with a small hook on their back. The front of the disks was painted with primer. The back was covered with felt (to minimize sounds that might have arisen from coming into contact with the crossbars). The largest disk (the reference disk) was 25.4 cm in diameter. The five comparison disks had diameters of 5.1 cm, 9 cm, 13.5 cm, 17.5 cm and 22.9 cm. The angular size differences between the reference and the comparison disks were approximately 31.6°, 26.4°, 19.8°, 13.5° and 4.3°.

#### Visual Stimulus

A pair of goggles (WolfBike X400 cycle goggles with clear lens, WolfBike Sports Goods, Guang Dong, China) was fitted with eight 3 mm white light emitting diodes (Kingbright, California, USA, part No. WP7104QWC/D). At 3 V, each LED had an average luminous intensity of 1.2 cd, 34 °. The LEDs were positioned on the inner rim of the frame (4 top/4 bottom), approximately 4.5 cm apart and parallel to the lens. The outer lens surface was painted black to block external light. The LEDs were wired to a portable, battery-powered (3 V) push button box.

#### Tactile Stimulus

We applied tactile stimulation using transcutaneous electrical nerve stimulation (TENS). This was administered using the Med-Fit Premier Plus TENS device (MedFit, Stockport, United Kingdom). The device produced a square wave (80 Hz, pulse width of 180 μs) eliciting the sensation of steady continuous vibration on the skin. For 10 sighted and the blind participants we used a pulse amplitude of 10 mA (based on results from the ‘Visual-Tactile Matching Experiment’). For the remaining 12 sighted participants we obtained a pulse amplitude that matched their perceived intensity of the visual stimulus (established using procedures as described in the ‘Visual-Tactile Matching Experiment’). The average setting for that group was 9.4 (SD: 3.4; min: 5; max 16) and this did not differ significantly from a setting of ‘10’ (t(11) = 0.586; p = 0.570; mean difference: −0.6; 95%CI: −2.78; 1.61). Participants wore two Med-Fit Premier Plus TENS self-adhesive electrodes each measuring 5 cm^2^ attached to the outside of their right forearm 5 cm apart with the centre of the first electrode placed 10 cm from the wrist joint towards the elbow.

### Task and Procedure

The experiment consisted of two sessions. All sighted participants were randomly assigned to the visual or tactile stimulation condition, and completed both sessions in their designated condition. Blind participants were assigned to the tactile condition and did only one session. The procedure for session one and two was identical. Any session took on average 1 hour 30 mins to complete.

#### Visual Stimulation

Participants wore the black-out goggles, and were instructed to keep their eyes open inside the goggles. In ‘on’ trials, visual interference was applied by pressing the button on the switch-box to trigger the small white LED lights to illuminate inside the goggles. In ‘off’ trials, the button was not pressed and participants completed the trial wearing the goggles, but with the LED lights switched off. There were an equal amount of ‘on’ and ‘off’ trials (60 trials of each per session), and the order was pseudorandomised. Specifically, stimuli were presented in blocks, so that every block of 20 trials contained two repetitions of each comparison disk size at each stimulation level. Within each block of 20 the order of disk sizes and stimulation levels was random.

#### Tactile Stimulation

Participants wore the black-out goggles (always ‘off’) and were instructed to keep their eyes open inside the goggles. Participants wore two electrodes attached to their right outer forearm. In ‘on’ trials, tactile interference was applied by running the current through the electrodes so that vibrations were felt. In ‘off’ trials electrodes were disconnected and no vibrations were felt. There were an equal amount of ‘on’ and ‘off’ trials (60 trials of each per session), and the order was pseudorandomised, just as for visual stimulation conditions.

#### Echolocation Task

Participants completed the echolocation task in line with the procedure of^7,8^. The general procedure for the echolocation task was the same for tactile and visual stimulation groups. Participants were seated on an adjustable chair 33 cm from the apparatus. The height of the chair was adjusted so that their ear was equidistant to the top and bottom crossbar of the apparatus. They were positioned square-on, directly in front of where the disks were going to be placed on the horizontal bars. The experimenter demonstrated an appropriate mouth-click before the participant was then asked to practise making similar clicks. When satisfactory mouth-clicks were produced by the participant, they completed two practice trials before the experiment commenced. There were a total of 120 trials, each following the same sequence. Participants blocked their ears with their respective left and right index fingers whilst the experimenter positioned the two disks on the crossbars. The (larger) reference disk was used in every trial, and placed either on the top or the bottom crossbar. It was placed on the top and bottom an equal amount of times (60 trials each). One of the five (smaller) comparison disks was placed on the remaining, free crossbar. Each comparison disk was used 24 times, positioned on the top crossbar and the bottom crossbar 12 times each. The TENS or goggles, dependent on condition, was then switched on (or remained off). It remained on/off for the duration of the trial. The participant was tapped on the shoulder to signal they should unblock their ears. The participant first made a no-click judgement: they simply indicated (with a silent hand signal) whether they believed the reference disk (larger disk) was on the top or bottom cross bar. The no-click judgements were a control, designed to measure whether any ambient noise, revealing information about the disk placement, was present. Following this judgement, another shoulder tap signalled that the participant should start making mouth clicks. They were given up to 14 seconds to make the clicks. If still making clicks at 14 seconds, a further shoulder tap was given to prompt a judgement. As with the no-click judgement, participants indicated whether they believed the reference disk was on the top or bottom crossbar with a silent hand signal.

### Data Analysis

Following previous work^7,8^, we calculated the proportion of correct answers for ‘no-click’ and ‘click’ judgments. Echolocation ability was then calculated by subtracting scores in ‘no-click’ conditions from those in ‘click’ conditions. For example, if a participant scored correct on every trial in ‘click’ conditions, and at chance (50%) in ‘no-click’ conditions, they would have an echolocation ability score of 0.5. Data from sighted participants were subsequently analysed using ANOVA, with ‘stimulation type’ (visual vs. tactile) as between-subjects variable, and ‘stimulation level’ (on vs. off), ‘disk size’ (comparison disks 1–5) and ‘session’ (1 vs. 2) as within-subjects variables. Greenhouse-Geisser (GG) correction was applied in cases where the sphericity assumption could not be upheld. We also tested if sighted participants’ scores in ‘tactile stimulation on’ conditions differed between those that had received pulse amplitude of 10 mA and those that had received individual settings. Furthermore, blind participants’ performance was compared to performance of the sighted sample in tactile conditions using non-parametric Mann-Whitney U-tests and t-tests adapted for comparison of single cases to a control sample^29^. Thresholds for statistical significance were p < 0.05 (two-tailed).

### Pre-Cursor Experiment - Visual-Tactile Matching Experiment

This experiment was a pre-cursor experiment to the main experiment. Specifically, in order to sensibly compare effects of visual and tactile stimulation in the main experiment, we first established that visual and tactile stimuli used were matched in terms of their ‘intensity’. Previous work has shown that people can reliably establish cross-modal visual-tactile intensity matches^30^, supporting the validity of this approach.

### Participants

To achieve a reliable estimate of average setting we assumed to need a minimum of 30 participants. A total of 36 sighted people participated (18 male; mean age: 28.0; min:19; max: 56; SD: 9.2). All participants reported to have normal hearing and no history of any hearing difficulties, and normal or corrected to normal vision. Participants volunteered to take part in the study and were compensated £7.50/hour or with participant pool credit.

### Set-Up and Apparatus

The experiment was conducted in the same room as the main experiment.

#### Visual Stimulus

The same goggles as in the main experiment were used, and participants were instructed to keep their eyes open inside the goggles. One modification was that the goggles could run at the intensity used in the main experiment, as well as at a lower intensity which was created using a 1 k Ohm resistor. The two settings were managed with a toggle switch.

#### Tactile Stimulus

The same TENS device and set-up as used in the main experiment was used.

### Task and Procedure

The experiment consisted of two sessions. On each trial the participant was simultaneously exposed to a visual stimulus and a tactile stimulus. The participant could be exposed to one of the two intensities of the visual stimulus and their task was to adjust the tactile stimulus until their perceived intensity of the tactile stimulation matched their perceived intensity of the visual stimulus. There were 24 trials in each session, with 12 presentations each of ‘high’ and ‘low’ visual settings in block-randomized order. For each trial the experimenter set the TENS starting value to a random intensity (within the range of 1 and 17 mA). Regardless of the starting value, the participant was free to adjust the TENS intensity however high or low in order to find their perceived match. The experimental instructions directed the participant to find a match between their perceived intensity of the two modalities by adjusting the TENS accordingly. Each session lasted about 30 minutes.

### Data Analysis

Participants’ settings for low and high luminance settings were averaged across trials within a session. We computed repeated measures ANOVA with ‘session’ (1 vs. 2) and ‘intensity’ (high vs. low) as factors. In order to test replicability of settings across sessions we used linear regression analyses. Threshold for statistical significance was set to p < 0.05 (two-tailed).

### Control Experiments – Effects of Visual Stimulation on Detection of Changes in Sound Frequency, Intensity and Location

The goal of these experiments was to determine if the effect of visual stimulation found in the main experiment was specific to echolocation, or if it would also apply to other auditory tasks. In this case we would expect to find a similar drop in performance between ‘on’ and ‘off’ conditions when people would, for example, have to detect a change in the intensity, frequency or location of a sound.

### Participants

Based on results from our main experiment (see ‘Results’ section, i.e. ‘visual stimulation off’ mean: 0.2047, SD: 0.1124; ‘visual stimulation on’ mean: 0.1271, SD: 0.1321; correlation between groups: 0.725) and using statistical power analysis software G-power 3.1^31^, we determined that we would need a minimum of 20 participants to achieve statistical power of 0.95. This was calculated based on alpha = 0.05 (two-tailed), and assuming that ‘on’ and ‘off’ would be a repeated measures factor.

A total of 59 sighted people participated. 26 participated in a test measuring detection of changes in sound frequency (DFM test) (5 male; mean age: 22.1; min: 19; max: 36; SD: 4.8), and 25 in a test measuring detection of changes in sound intensity (DCI test) (8 male; mean age: 26.8; min:19; max: 42; SD: 6.8). 22 participated in a test measuring detection of changes in sound location (10 male; mean age: 29.4; min: 19; max: 58; SD: 8.8). 12 of the participants took part in both DCI and DFM tests. One of the participants took part in DCI, DFM and localization tests. All participants reported to have normal hearing and no history of any hearing difficulties, and normal or corrected to normal vision. Participants volunteered to take part in the study and were compensated £7.50/hour or with participant pool credit.

### Set-Up and Apparatus

The experiment was conducted in the same room as the main experiment.

#### Visual Stimulus

The same stimulus as in the main experiment was used.

#### Auditory Testing

Auditory testing was conducted using an IBM Lenovo N500 laptop (Intel Pentium Dual PCU T3400 2.16 GHz, 3 GB RAM, 64 bit Windows 7 Enterprise SP1). Software used to conduct testing was programmed using Psychophysics Toolbox 3.0.8^32^ and Matlab (R2013b, The Mathworks, Natick, MA, USA). Sounds were presented using a Creative Sound Blaster X-Fi HD Sound Card (Creative Technology Ltd., Creative Labs Ireland, Dublin, Ireland) and AKG K271 MKII Circumaural Studio Headphones (Harman International Industries, Stamford, CT, USA).

### Task and Procedure

Each experiment consisted of one session. Those participants who took part in only one test were randomly assigned to a session. For participants who took part in more than one test, we counterbalanced the order in which tests were presented. Any session took between 1 and 1.5 hours to complete.

#### Detection of Changes in Sound Frequency

This test was a replication of the test used by^8,33^. The test measures participant’s ability to detect a change in the frequency (pitch) of a tone. On each trial participants were presented with a pair of tones, and they pressed a button whenever they detected a change in frequency in the second tone of a pair. Each pair consisted of a 2-second steady pure tone (incl. 80 ms linear on and off ramp), followed by another 2-second tone (incl. 80 ms linear on and off ramp), that could be either another steady pure tone (‘catch-trial’), or a frequency modulated tone. There was a 300 ms silent gap in between the two tones. The frequency modulation was 300 ms long. To avoid participants predicting when a frequency modulation would occur, the onset time of the modulations was randomized, with the limitation that modulations could only occur after an 800 ms lead-in of the continuous tone. Participants were informed that the first tone was always a steady tone, and they were told that they could use this as a reference for assessing any changes in the second tone. We used three increments of frequency modulation (0.6, 0.4, 0.2 percent modulation) and 18 tests were presented for each increment. Tests were conducted at centre frequencies of 500 and 2000 Hz. Test trials were preceded by a practice series with increments in frequency modulation that gradually decreased from 5 to 2 percent. Following^33^ the test was presented at 500 Hz and 2000 Hz at 30 dB Hearing Threshold (HT) (as determined for our set-up; a single HT value was obtained for each participant by averaging HT across the right and left ears).

#### Detection of Changes in Sound Intensity

This test was a modified replication of the test used by^8,33^. The test structure was the same as for the frequency test described above, with the only difference that the tone is modulated in intensity (loudness) instead of frequency. We used three increments of intensity modulation (1.2, 1.0 and 0.8 dB) and 18 tests were presented for each increment. There were also 18 catch trials. Tests were conducted at centre frequencies of 500 and 2000 Hz. Participants were instructed to press a button whenever they detected a jump in loudness. Test trials were preceded by a practice series with increments in intensity that gradually decreased from 5 dB to 2 dB. Following^33^, the test was presented at 500 and 2000 Hz at 45 and 35 dB Hearing Threshold (HT), respectively (as determined for our set-up; a single HT value was obtained for each participant by averaging HT across the right and left ears).

The intensity test used here was a modified replication of a test used previously^8,33^. The reason we had modified it was that performance in previous instalments was comparably poor (i.e. many people performed at chance). Thus, in order to avoid floor effects we changed the test from a single interval presentation to a two-interval presentation (i.e. we now always presented a ‘reference sound’ first). This also makes this test more similar to the test used to measure detection of changes in sound frequency.

#### Detection of Changes in Sound Location

The test used was a modified replication of a test we have used previously^34^. Briefly, on each trial participants were presented with two sounds in two separate intervals. The first sound was always the reference sound (located 0° straight ahead), whilst the second sound could be shifted either to the right or to the left from straight ahead (20°, 10°, 5°, 2.5°, 1.5° and 0.5° to the left and right of straight ahead; all in the horizontal meridian). The participant’s task was to indicate via button press if the second sound was located to the left or to the right of the first sound. Sounds were computer generated (44.1 kHz, 16 bit) using the Super Collider audio programming language. Sounds were 0.5–10 kHz bandpass filtered white noise with a 40-Hz sinusoidal amplitude modulation (between zero and maximum amplitude) and of 1 s duration. HRTF filter coefficients were derived from a set of measurements conducted with a Knowles Electronic Mannequin for Acoustic Research (KEMAR) under anechoic conditions^35^. Sounds were presented at approximately 60 dB SPL. There were 10 repetitions for each testing location and luminance condition, thus 240 trials total. Trials were presented in random order.

#### Visual Stimulation

Participants wore the same black-out goggles as in the main experiment and were instructed to keep their eyes open inside the goggles. In ‘on’ trials, visual interference was applied by pressing the button on the switch-box to trigger the LED lights to illuminate inside the goggles. In ‘off’ trials, the button was not pressed and participants completed the trial wearing the goggles, but with the LED lights switched off. There were an equal amount of ‘on’ and ‘off’ trials per session and test, and the order was randomised.

### Data Analysis

#### Detection of Changes in Sound Frequency and Intensity

Catch trials were used to calculate proportion of false alarms. For each test and participant we then subtracted proportion of false alarms from proportion of correct detections for ‘on’ and ‘off’ conditions separately. We then subjected these data to repeated measures ANOVA with variables ‘frequency’ (2000 vs. 500 Hz) and ‘visual stimulation’ (on vs. off) for Intensity and Frequency tests separately. Threshold for statistical significance was set to p < 0.05 (two-tailed).

#### Detection of Changes in Sound Location

Data were used to calculate proportion of ‘right’ judgments for each location and visual stimulation condition. We then fitted two-parameter sigmoid curves of the form $F=\frac{1}{1+\exp (-\frac{x-a}{b})}$ to data for each luminance condition separately (using a non-linear least squares fit implemented in matlab optimization toolbox). To compute thresholds we first determined those points on the curve where the probability to judge a stimulus as right was either 0.25 or 0.75. We then computed the average of the absolute threshold values. We then compared thresholds between ‘on’ and ‘off’ conditions using paired t-tests. Threshold for statistical significance was set to p < 0.05 (two-tailed).

### Data Availability

All data generated or analysed during this study are included in this published article (and its Supplementary Information files).

## Results

### Main Experiment - Effects of Visual and Tactile Sensory Stimulation on Echolocation of Size

Repeated measures ANOVA showed that sighted participants’ echolocation scores in no-click conditions did not differ from chance (i.e. 0.5) (t(43) = 0.08; p = 0.937; mean score: 0.5; 95%CI: 0.489; 0.511), and that performance was unaffected by ‘stimulation type’ (visual vs. tactile), ‘stimulation level’ (on vs. off), ‘session’ (1 vs. 2), or ‘disk size’ (comparison disks 1–5), i.e. none of the main effects or interactions were significant (data provided in Supplementary Table S1). In conclusion, as expected participants performed the same across all ‘no-click’ conditions and their performance in ‘﻿no-click’ conditions﻿ was at chance level.

Sighted participants’ echolocation scores in ‘tactile stimulation on’ conditions did not differ between those that had received pulse amplitude of 10 mA and those that had received individual settings (t(20) = 0.119; p = 0.636; mean difference: 0.007; 95%CI: −0.13; 0.11). Also, the mean difference between ‘off’ and ‘on’ conditions did not differ between these two groups (t(20) = −0.316; p = 0.755; mean difference: −0.013; 95%CI: −0.095; 0.070). Thus, we did not dissociate between these two groups for further analyses.

**Figure 2:**
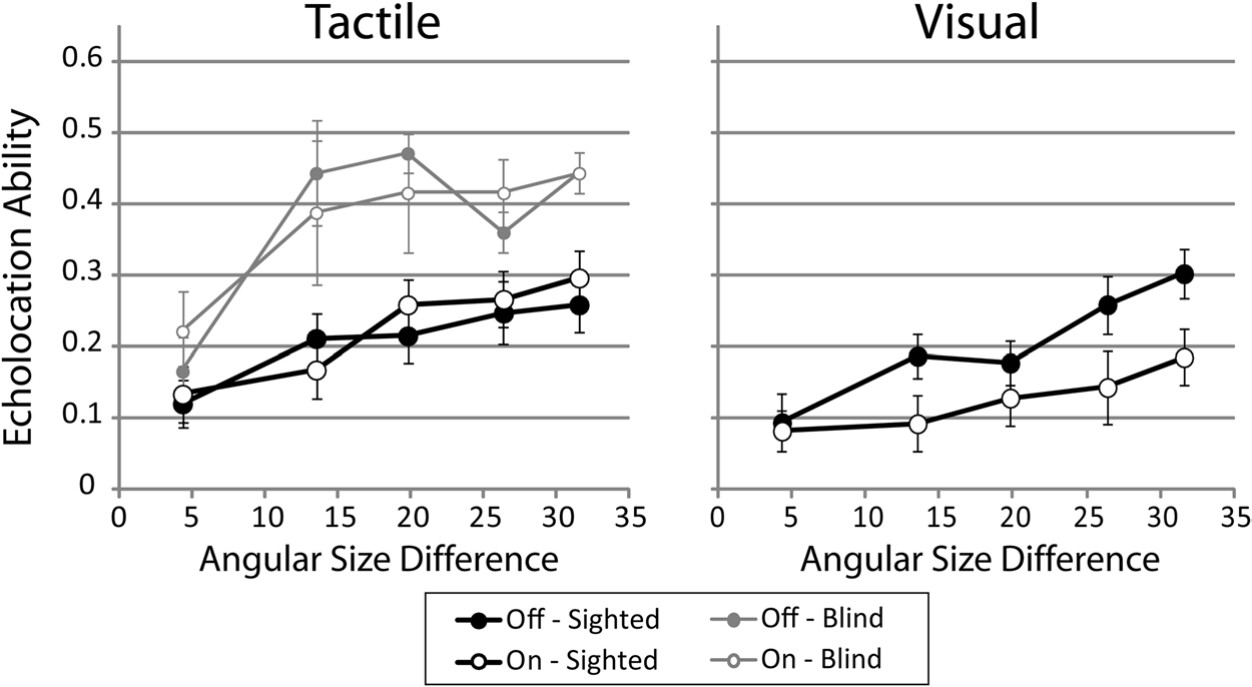
Visual sensory stimulation (but not tactile sensory stimulation) impairs people’s ability to echolocate. Data for sighted and blind participants are plotted in black and grey lines, respectively. Tactile– Tactile sensory stimulation. Visual– Visual sensory stimulation. Filled and open symbols denote stimulation ‘off’ and ‘on’ respectively. Data are plotted as a function of the angular size difference between the reference and comparison disks. The reference disk was 25.4 cm in diameter. The five comparison disks had diameters of 5.1 cm, 9 cm, 13.5 cm, 17.5 cm and 22.9 cm, resulting in angular size differences between the reference and the comparison disks of approximately 31.6°, 26.4°, 19.8°, 13.5° and 4.3°. Symbols are means and error bars are standard errors across participants.

Figure 2 shows data separately for the various disk sizes and interference conditions. Data from sighted participants with no prior experience in echolocation are plotted in black lines, and data from blind participants with experience in echolocation in grey lines. Repeated measures ANOVA applied to data from sighted participants showed a significant effect of ‘disk size’ (F_GG_(3.161, 132.776) = 13.421; p < 001; η^2^_p_ = 0.242), with a significant linear trend (F(1,42) = 31.435; p < 0.001; η^2^_p_ = 0.428). In conjunction with Fig. 2 this demonstrates that performance increased as the size difference between reference and comparison disk became larger. Furthermore, the effect of ‘stimulation level’ was significant (F(1,42) = 5.313; p = 0.026; η^2^_p_ = 0.112), and the interaction effect between ‘stimulation type’ and ‘stimulation level‘ was also significant (F(1,42) = 11.030; p = 0.002; η^2^_p_ = 0.208). None of the other effects were significant (data provided in Supplementary Table S2).

**Figure 3:**
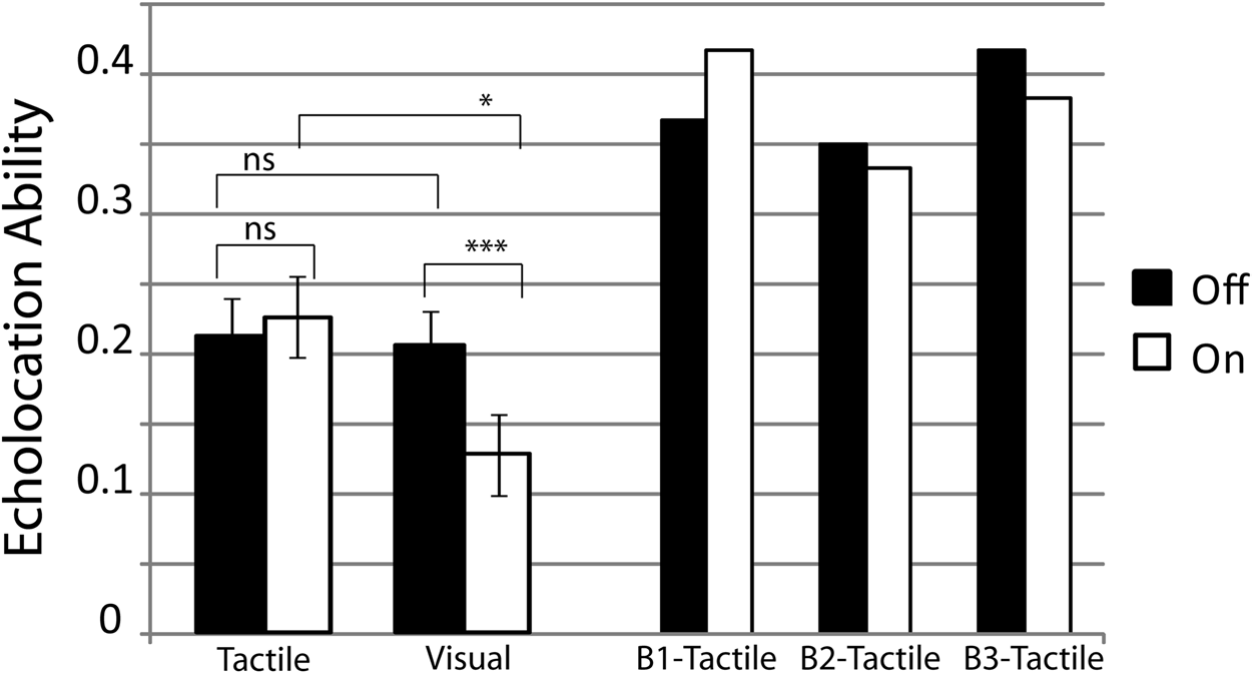
Visual sensory stimulation (but not tactile sensory stimulation) impairs people’s ability to echolocate. Left hand side: Performance of sighted participants (N = 44). Tactile– Tactile sensory stimulation. Visual– Visual sensory stimulation. Bars indicate averages across participants, error bars indicate standard errors across participants. Right hand side: Performance of blind echo-expert participants B1–B3 (narrow bars), who only took part in tactile conditions. *p < 0.05; **p < 0.01; ***p < 0.001; ns = non-significant

Therefore, we averaged echolocation ability scores across disk sizes and sessions to further investigate the significant interaction effect. Figure 3 shows data averaged across disk sizes from sighted participants with no prior experience in echolocation (wide bars, left hand side), and from blind participants with experience in echolocation (narrow bars, right hand side).

Follow up t-tests showed that performance dropped significantly (t(21) = 3.933; p = 0.001; correlation: 0.725; 95%CI: 0.0366; 0.119; paired samples test) when visual stimulation was ‘on’ (mean: 0.127, SD: 0.132) as compared to when it was ‘off’ (mean: 0.205, SD: 0.112). In contrast, there was no change in performance (t(21) = −0.727; p = 0.475; correlation: 0.751; 95%CI: −0.054; 0.026; paired samples test) when tactile stimulation was ‘on’ (mean: 0.225, SD: 0.13) or ‘off’ (mean: 0.211, SD: 0.126). Performance in ‘off’ conditions did not differ between tactile and visual stimulation (t(42) = 0.186; p = 0.854; 95%CI: −0.0658; 0.0792; independent samples t-test), but it differed in ‘on’ conditions (t(42) = 2.482; p = 0.017; 95%CI: 0.0184, 0.1782; independent samples t-test).

Average performance of blind participants was the same when tactile stimulation was switched ‘on’ (mean: 0.378; SD: 0.042) or ‘off’ (mean: 0.378, SD: 0.035). To determine for each blind participant if their performance was affected by tactile stimulation being ‘on’ or ‘off, we computed the Revised Standardized Difference Test (RSDT)^29^. This test determines if the difference between an individual’s score in two conditions/tasks (here tactile stimulation being ‘on’ or ‘off’) is significantly different from the differences observed in a control sample. The result of this test was not significant for any of our blind participants (B1: t(21) = 0.317; p = 0.7543; B2: t(21) = 0.362; p = 0.7212; B3: t(21) = 0.557; p = 0.5834). Thus, effects of tactile stimulation are the same regardless of people’s sensory status (sighted vs. blind) or experience with echolocation (no experience vs. experience). As expected, however, blind echolocation experts did perform significantly better than sighted participants in tactile interference conditions (Mann Whitney U test, U(25) = 6; p = 0.024; Sighted (n = 22) mean rank: 11.77; Blind (n = 3) mean rank: 22).

### Pre-Cursor Experiment - Visual-Tactile Matching

We conducted this experiment in order to establish that visual and tactile stimuli used in the main experiment were matched in terms of their ‘intensity’. We used both a ‘high’ and a ‘low’ luminance condition, and we tested participants in two separate sessions. Previous work has shown that people can reliably establish cross-modal visual-tactile intensity matches^30^, supporting the validity of this approach.

There was a significant effect of luminance (F (1,35) = 188.35, p < 0.001; η^2^_p_ = 0.843). High-luminance yielded a significantly greater TENS value (*M* = 9.5, *SD* = 3.1) as compared to low-luminance (*M* = 4.9, *SD* = 2.3). The effect of session was non-significant (F (1,35) = 0.000, p = 0.984; η^2^_p_ = 0.000), with a mean TENS intensity value of 7.2 (*SD* = 2.7) and 7.2 (*SD* = 2.6) for S1 and S2, respectively. The interaction between luminance and session was also non-significant (F (1,35) = 1.525, p = 0.225; η^2^_p_ = 0.042). Responses to high-luminance returned an average TENS intensity value of 9.4 (*SD* = 3.2) in S1 as compared to 9.6 (*SD = *3.2) in S2. Responses to low-luminance returned an average TENS intensity value of 4.9 (*SD* = 2.5) in S1 and 4.8 (*SD* = 2.3) in session 2.

Linear regression showed that settings in session 1 were a reliable predictor of settings in session 2 (High luminance conditions: r = 0.95; r^2^: 0.89; constant: 0.706 [95%CI: −0.421; 1.833]; t(34) = 1.272; p = 0.212; slope: 0.939 [95%CI: 0.826; 1.053]; t(34) = 16.845; p < 0.001; Low luminance conditions: r = 0.84; r^2^: 0.71; constant: 0.996 [95%CI: 0.045; 1.947]; t(34) = 2.128; p = 0.041; slope: 0.772 [95%CI: 0.600; 0.944]; t(34) = 9.111; p < 0.001). Residuals were normally distributed (high luminance conditions: SW(36) = 0.956; p = 0.166; low luminance conditions: SW(36) = 0.981; p = 0.767).

The regression analysis shows that participants were consistent in their matches across sessions, in particular for high-luminance. The average TENS intensity value for high luminance settings across sessions 1 and 2 was 9.5 (SD = 3.1) (and this was normally distributed, SW (36) = 0.991; p = 0.987). Based on these results we chose a TENS intensity value of 10 mA for the main experiment.

### Control Experiments - Effects of Visual Stimulation on Detection of Changes in Sound Frequency, Intensity and Location

The goal of these experiments was to determine if the effect of visual stimulation we had found in the main experiment, was specific to echolocation, or if it would also apply to other auditory tasks. In this case we would expect to find a similar drop in performance between ‘on’ and ‘off’ conditions when people would for example have to detect a change in the intensity, frequency or location of a sound.

#### Detection of Changes in Sound Frequency

**Figure 4:**
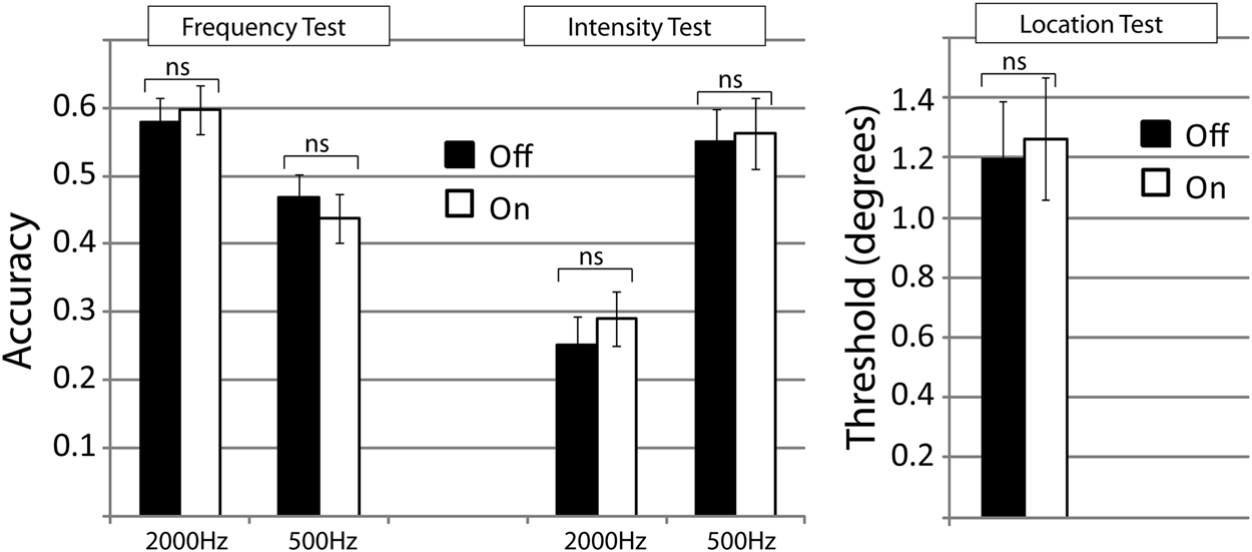
Visual sensory stimulation has no effect on people’s ability to detect changes in sound frequency, intensity or location. Performance of sighted participants in auditory control experiments: Frequency (n = 26) and Intensity (n = 25) discrimination conducted at both 2000 and 500 Hz, and Location discrimination (n = 22). Bars indicate averages across participants; error bars indicate standard errors across participants. *p < 0.05; **p < 0.01; ***p < 0.001; ns = non-significant

The effect of ‘frequency’ was significant (F(1,25) = 22.730; p < 0.001; η^2^_p_ = 0.476), with participants having performed better in 2000 Hz (mean: 0.57; SD: 0.17) as compared to 500 Hz conditions (mean: 0.43; SD: 0.18). The effect of ‘visual stimulation’ was non-significant (F(1,25) = 0.068; p = 0.796; η^2^_p_ = 0.003). The interaction effect was non-significant, too (F(1,25) = 1.87; p = 0.184; η^2^_p_ = 0.070). Results are illustrated in Fig. 4. Performance in the frequency task agrees with performance we found in previous work^8^.

#### Detection of Changes in Sound Intensity

The effect of ‘frequency’ was significant (F(1,24) = 31.380; p < 0.001; η^2^_p_ = 0.567), with participants having performed worse in 2000 Hz (mean: 0.27; SD: 0.16) as compared to 500 Hz conditions (mean: 0.56; SD: 0.23). The effect of ‘visual stimulation’ was non-significant (F(1,24) = 0.530; p = 0.474; η^2^_p_ = 0.022). The interaction effect was non-significant, too (F(1,24) = 0.242; p = 0.627; η^2^_p_ = 0.010). Results are illustrated in Fig. 4. Performance in the Intensity task was better as compared to what we found previously^8^, likely due to the fact that we had changed the format of the task to avoid floor effects.

#### Detection of Changes in Sound Location

The effect of ‘visual stimulation’ on localization thresholds was non-significant (t(21) = 0.437; p = 0.666; correlation: 0.743; 95%CI of the difference: −0.401; 0.278). Results are illustrated in Fig. 4.

In sum, there was no effect of visual stimulation in any of the control tasks.

## Discussion

Sighted participants’ echolocation performance decreased when visual stimulation was provided. In contrast, tactile stimulation had no effect on echolocation performance in sighted or blind people. Blind participants performed better overall, which was expected considering their experience in echolocation. The results from our visual-tactile matching task showed that TENS values chosen yielded an intensity of tactile stimulation that matched the intensity of visual stimulation. Thus, visual and tactile stimulation levels had been matched in terms of their perceived intensity, ruling out the possibility that the tactile stimulation we had used was not strong enough to have had any effect. Furthermore, we showed that the visual stimulation did not have any effect on performance in auditory control tasks that required detection of changes in sound intensity, frequency or location. This demonstrates that the effect of visual stimulation was specific to echolocation in our experiment, and rules out an attention based explanation. Taken together, the results provide behavioural evidence suggesting that even in sighted people echolocation and vision share neural resources.

Previous neuroimaging work has shown that people who are blind and who have experience in echolocation show activation in ‘visual’ brain areas when processing echolocation sounds, whilst sighted people did not show any echolocation related activity in visual areas^11–15^. This could have been due to lack of statistical power, or lack of echolocation skill, or both. The current study used a behavioural paradigm, which may have been more sensitive to interactions between vision and echolocation in sighted people. Based on our results we would predict that future neuroimaging studies, using more skilled samples and/or increased statistical power, might find echolocation related activity in echolocating sighted people’s brains within areas that are traditionally associated with ‘vision’, e.g. calcarine cortex.

We previously found that sighted people’s ability to echolocate size correlated positively with their score on a mental imagery task (VVIQ)^8^. Neuroimaging has shown that activity in calcarine cortex is correlated with mental imagery as measured with VVIQ in sighted people^36^. We cannot rule out the possibility that any effects of visual stimulation on echolocation might be mediated by effects of visual stimulation on mental imagery^28^. Nonetheless, this idea would still require echolocation to draw on sensory visual processing resources.

There is other research suggesting that ‘visual’ brain areas, including calcarine cortex, can be involved in processing information from other modalities. Much of this research is based on work with people who are blind, so that the observed functionality might be due to neuroplastic changes arising in response to long term deprivation^16–23^. Nonetheless, some of these results have also been reported in people who are sighted, suggesting that long-term reorganization might not be necessary for ‘visual’ brain areas to respond to information from other modalities^37^. Importantly, however, results accumulating from research in both blind and sighted people suggest strongly that it is not the modality itself that determines the presence or absence of any interactions, but the task or task-conditions within a modality. With reference to auditory-visual interactions in people, it has been shown that TMS to right middle occipital gyrus in early blind people interfered with processing of spatial sound-location, but not with processing of pitch and intensity^38^. Notably, however, the same study did not find any effects of TMS in people who are sighted. With respect to visual-tactile interactions, it was found in a sample of sighted people that TMS over right extrastriate cortex lead to an impairment in discrimination of orientation of tactile gratings, but did not affect discrimination of surface roughness^39^. Results that propose similar within-modality specificity have also been reported in profoundly deaf cats, and changes in performance in visual tasks. For example, it has been shown that cooling of certain areas within auditory cortex in congenitally deaf cats may affect their performance in visual localization or visual motion detection, but not in tasks that measure visual Vernier acuity, or orientation discrimination^40^. Based on these results it has been suggested that only those aspects of processing that could transfer across modalities might be associated with cross-modal neural changes^40^. These, and similar ideas^41^ might provide a useful framework for future investigations into the anatomical and computational links across modalities.

## Electronic supplementary material

Supplementary Materials
