# Supplementary material for: Visual sensory stimulation interferes with people’s ability to echolocate object size
Source: Sci Rep. 2017 Oct 12;7:13069. doi: 10.1038/s41598-017-12967-3 (PMC5638915; doi:10.1038/s41598-017-12967-3)
Supplement: Supplementary file 1 — Supplementary Materials [file 41598_2017_12967_MOESM1_ESM.pdf]

Visual sensory stimulation interferes with people's ability to echolocate object size

-- Supplementary Materials --

Thaler[1], L. & Foresteire[1], D.

1- Department of Psychology, Durham University, Durham, United Kingdom

Corresponding author:

Lore Thaler
lore.thaler@durham.ac.uk
Department of Psychology, Durham University
Science Site, South Road
Durham DH1 3LE
United Kingdom

Supplementary Table S1 – Results of ANOVA analysis applied to sighted participants echolocation scores in 'no click' conditions

**Effect**	**F**	**p**	η^2_p
Session	F(1,42)=.796	.377	.019
Session x stimulation type	F(1,42)=.580	.451	.014
Disk size	F(4,168)=.533	.712	.013
Disk size x stimulation type	F(4,168)=.382	.821	.009
stimulation level	F(1,42)=.245	.623	.006
stimulation level x stimulation type	F(1,42)=1.885	.177	.043
Session x disk size	F(4, 168)=.299	.878	.007
Session x disk size x stimulation type	F(4, 168)=2.258	.065	.051
Session x stimulation level	F(1,42)=.851	.362	.020
Session x stimulation level x stimulation type	F(1,42)=1.204	.279	.028
Disk size x stimulation level	F(4, 168)=.351	.843	.008
Disk size x stimulation level x stimulation type	F(4,168)=.624	.646	.015
Session x disk size x stimulation level	F_{GG}(3.376,141.772)=.775	.523	.018
Session x disk size x stimulation level x stimulation type	F_{GG}(3.376,141.772)=1.080	.368	.025
Stimulation type	F(1,42)=.073	.788	.002

Supplementary Table S2 – Results of ANOVA analysis applied to sighted participants echolocation ability scores.

**Effect**	**F**	**p**	η^2_p
Session	F(1,42)=3.809	.058	.083
Session x stimulation type	F(1,42)=1.166	.286	.027
Disk size	F_{GG}(3.161, 132.776)=13.421	<.001	.242
Disk size x stimulation type	F(4,168)=.368	.831	.009
stimulation level	F(1,42)=5.312	.026	.112
stimulation level x stimulation type	F(1,42)=11.030	.002	.208
Session x disk size	F(4, 168)=2.112	.082	.048
Session x disk size x stimulation type	F(4, 168)=1.436	.224	.033
Session x stimulation level	F(1,42)=1.553	.220	.036
Session x stimulation level x stimulation type	F(1,42)=.466	.498	.011
Disk size x stimulation level	F(4, 168)=1.134	.342	.026
Disk size x stimulation level x stimulation type	F(4,168)=.935	.445	.022
Session x disk size x stimulation level	F_{GG}(3.384,142.148)=.495	.708	.012
Session x disk size x stimulation level x stimulation type	F_{GG}(3.384,142.148)=.771	.526	.018
Stimulation type	F(1,42)=2.222	.144	.050
